# S199. ENHANCED OLFACTORY IDENTIFICATION IN ADOLESCENTS WITH PSYCHOTIC EXPERIENCES

**DOI:** 10.1093/schbul/sby018.986

**Published:** 2018-04-01

**Authors:** Eleanor Carey, Niamh Dooley, Erik O’Hanlon, Ian Kelleher, Mary Cannon

**Affiliations:** Royal College of Surgeons in Irel

## Abstract

**Background:**

The olfactory system has a widely distributed anatomical network reaching both cortical and subcortical structures (Milardi et al., 2017). Olfactory dysfunction has been associated with schizophrenia (Moberg & Turetsky, 2003), where deficits in odour identification (Seidman et al., 1997), odor detection threshold sensitivity (Serby et al., 1990) and odour memory (Wu et al., 1993) can be seen early in the course of the disorder and persist with illness duration (Rupp, 2010). This olfactory dysfunction has been correlated with cognitive deficits, including language (Corcoran et al., 2005), verbal and non-verbal memory (Moberg et al., 2006) and tests of emotional recognition (Goudsmit et al., 2003) in schizophrenia. Neuroanatomically, areas of the temporal lobe, including the superior temporal gyrus, hippocampus and amygdala have all been implicated in the dysfunction. It is not known whether olfactory changes can be detected in the broader extended psychosis phenotype.

**Methods:**

The current research focuses on a community-based sample of young adolescents aged 11–13 (N= 140) recruited from schools in the Dublin and Kildare areas of Ireland, These adolescents were assessed for psychotic symptoms using the psychosis section of the Schedule for Affective Disorders and Schizophrenia and also completed the Brief Smell Identification Test (BSIT), derived from the University of Pennsylvania Smell Identification Test (UPSIT),as part of a neuropsychological assessment. The BSIT is a self-administered 12-item test of olfactory functioning, containing common odours (eg lemon, chocolate, and smoke) and participants were required to choose one of four multiple-choice answers.

**Results:**

Performance on the BSIT was compared between participants who reported any psychotic experiences (PE) (N=71), and those who did not (N=69). We examined total score and scores for individual smell types, using ANOVA, and co-varied for age and gender.

**Results:**

A significant group difference was found when both age and gender were co-varied for, in which the PE group performed significantly better on the BSIT (F=5.56, p= 0.02), and one of the individual twelve odours (‘‘paint- thinner’’) of the BSIT also was significantly better identified by the PE group (F=7.53, p=0.007). When examined in terms of correlations with psychotic symptoms, BSIT scores were found to significantly correlate with Grandiosity, one of the eight categories of the Adolescent Psychotic-Like Symptoms Screener (APSS) (p=0.004).

**Discussion:**

Moberg et al. (2013) report that positive symptoms of psychosis may be moderating our results, where it was hypothesized that this early olfactory hypersensitivity is due to increased vigilance of external and internal stimuli. Corcoran et al. (2005) similarly found that the presence of features associated with mania, such as grandiosity, was associated with better outcomes in smell identification tests.

We hope to extend our analysis to search for neuroanatomical and neuropsychological correlates of these olfactory performance scores in young people with and without psychotic symptoms, as well as looking more in-depth at positive symptoms reported by the PE group and their possible associations with this increased hypersensitivity in olfactory identification. The current research is hoped to capture some of the earliest olfactory changes associated with psychosis vulnerability as a possible biomarker. The study employs a novel participant cohort whom are considered an extended psychosis phenotype and at this point only exhibit psychotic symptoms, but remain at an increased risk of the development of psychosis in adulthood.

